# Supplementary material for: The Effect of Rotavirus Vaccine on Socioeconomic Differentials of Paediatric Care Due to Gastroenteritis in Swedish Infants
Source: Int J Environ Res Public Health. 2019 Mar 27;16(7):1095. doi: 10.3390/ijerph16071095 (PMC6479834; doi:10.3390/ijerph16071095)
Supplement: Supplementary file 1 [file ijerph-16-01095-s001.pdf]

Table S1. Cox regression of the socioeconomic covariates related to paediatric inpatient and outpatient care of viral gastroenteritis in Jönköping County Council.

Socioeconomic covariates:	**Jönköping County Council** Difference-in-Differences estimate of HR (95 % CI)	
After vs. *Before* Vaccine start (*reference in italic*)	**Inpatient care for gastroenteritis**	**Outpatient care for gastroenteritis**
Maternal primary education vs. *Tertiary education*	1.26 (0.63-2.50)	1.06 (0.73-1.53)
Maternal secondary education vs. *Tertiary education*	**0.12** (0.02-0.90)	0.72 (0.43-1.21)
Paternal primary education vs. *Tertiary education*	1.05 (0.54-2.04)	1.11 (0.73-1.42)
Paternal secondary education vs. *Tertiary education*	0.41 (0.14-1.20)	1.13 (0.51-1.22)
Maternal age<25 at birth vs. *Other*	1.46 (0.75-2.82)	**1.45** (1.06-1.99)
Use social welfare benefit vs. *Other*	1.53 (0.70-3.33)	1.47 (1.00-2.17)
Both parents foreign born vs. *At least one Swedish-born parent*	1.26 (0.70-2.29)	1.03 (0.77-1.39)
Single parenthood household vs *Cohabitation*	1.27 (0.58-2.81)	1.45 (0.96-2.19)

Bold text indicates statistical significant associations on the p <0.05 level.

Table S2. The study population of the sensitivity analysis. N=519,128.

	**Stockholm County Council***		**All other Swedish county councils***	
	Born Before Vaccine (N= 83,031) (%)	Born After Vaccine (N= 52,900) (%)	Born Before Vaccine (N=235,287) (%)	Born After Vaccine (N=147,910) (%)
**Outcome**				
Inpatient care gastroenteritis	2.4	0.8	1.8	1.1
Outpatient care gastroenteritis	9.6	6.5	6.2	5.0
**Child characteristics**				
Sex				
Boy	51.4	51.8	51.4	51.6
Girl	48.6	48.2	48.6	48.4
Birth year				
2009	26.2	0.0	27.9	0.0
2010	34.8	0.0	33.9	0.0
2011	33.6	0.0	32.8	0.0
2012	5.4	0.0	5.4	0.0
2013	0.0	0.0	0.0	0.0
2014	0.0	46.2	0.0	45.6
2015	0.0	53.8	0.0	54.4
Preterm				
Yes	5.4	5.3	5.9	5.6
**Family characteristics**				
Maternal age at birth of child				
<25 years	8.1	7.0	14.4	12.8
25 or older	91.9	93.0	85.7	87.2
Maternal education				
Primary education	12.2	10.7	13.5	13.9
Secondary education	8.2	7.2	8.3	7.1
Tertiary education	79.6	82.1	78.2	79.0
Paternal education				
Primary education	15.7	16.0	15.3	16.7
Secondary education	12.4	9.8	15.6	11.5
Tertiary education	71.9	74.2	69.2	71.8
Use of social welfare benefit				
Yes	5.1	3.1	9.5	8.6
Cohabitation of parents in early pregnancy				
No	11.9	12.8	9.5	10.2

Parental country of birth				
Both parents foreign born	20.7	23.6	84.5	80.3
At least one Swedish-born parent	79.4	76.4	15.5	19.7

* The “Born Before Vaccine” group consisted of children born from March 2009-February 2012. The "Born After Vaccine" group consisted of children born from March, 2014.

Table S3. Sensitivity analysis of Cox regression of paediatric inpatient and outpatient care of viral gastroenteritis.

After vs. *Before* Vaccine start: (*reference in italic*)	Difference-in-Differences estimate of HR (95 % CI)
**Inpatient care for gastroenteritis**	
Stockholm vs *Rest of Sweden**	**0.64** (0.57-0.71)
**Outpatient care for gastroenteritis**	
Stockholm vs *Rest of Sweden**	**0.92** (0.88-0.96)

* The “Born Before Vaccine” group consisted of children born from March 2009-February 2012. The "Born After Vaccine" group consisted of children born from March, 2014.

Table S4. Sensitivity analysis of Cox regression of the socioeconomic covariates related to paediatric inpatient and outpatient care due to viral gastroenteritis.

Socioeconomic covariates:	Difference-in-Differences estimate of HR (95 % CI)	
After vs. *Before* Vaccine start (*reference in italic*)	**Inpatient care for gastroenteritis**	**Outpatient care for gastroenteritis**
	**Stockholm County Council***	
Maternal primary education vs. *Tertiary education*	1.00 (0.76-1.32)	**1.25** (1.12-1.39)
Maternal secondary education vs. *Tertiary education*	**1.44** (1.06-1.97)	**1.15** (1.02-1.31)
Paternal primary education vs. *Tertiary education*	1.16 (0.91-1.47)	**1.18** (1.07-1.29)
Paternal secondary education vs. *Tertiary education*	0.96 (0.69-1.32)	**1.14** (1.01-1.28)
Maternal age<25 at birth vs. *Other*	0.95 (0.67-1.34)	**1.30** (1.15-1.46)
Use social welfare benefit vs. *Other*	1.23 (0.80-1.89)	**1.39** (1.17-1.64)
Both parents foreign born vs. *At least one Swedish-born parent*	0.95 (0.76-1.17)	**1.15** (1.06-1.25)
Single parenthood household vs *Cohabitation*	1.00 (0.78-1.29)	1.10 (1.0-1.23)

* The “Born Before Vaccine” group consisted of children born from March 2009-February 2012. The "Born After Vaccine" group consisted of children born
